# The Impact of Thyroid Surgery on Calcium and Magnesium Homeostasis: A Prospective Study

**DOI:** 10.7759/cureus.67100

**Published:** 2024-08-17

**Authors:** Iqbal M Ali, Sarthak Sharma, Varun Shetty

**Affiliations:** 1 Department of General Surgery, Dr. D. Y. Patil Medical College, Hospital and Research Centre, Dr. D. Y. Patil Vidyapeeth (Deemed to be University), Pune, IND

**Keywords:** intra-capsular dissection, homeostasis, hypomagnesemia, hypocalcemia, total thyroidectomy

## Abstract

Objective: Total thyroidectomy constitutes one of the bread-and-butter procedures of surgeons all over the world. Like with any surgical procedure, complications form a part and parcel of the postoperative course in the hospital. Hypocalcemia represents one such prevalent complication post-total thyroidectomy. This study aimed to evaluate the impact of total thyroidectomy on calcium and magnesium levels and to assess the role of magnesium in postoperative hypocalcemia.

Methods and materials: This study was carried out at a tertiary health center over a two-year period from 2022 to 2024. It involved 100 participants with thyroid conditions (benign/malignant) who required total thyroidectomy. Patients with pre-existing conditions affecting calcium levels (e.g., chronic renal failure, medullary carcinoma thyroid, etc.) were expressly excluded. Preoperative calcium, magnesium, and parathyroid hormone (PTH) levels were recorded. Intraoperative parameters such as time and fluid volume were also measured. Postoperatively, serum calcium and magnesium levels, PTH levels, and complications like hypocalcemia and hypomagnesemia were monitored. The descriptive statistics were computed to delineate the study sample. After completion of data collection, data analysis was achieved using IBM SPSS Statistics for Windows, V. 16.0 (SPSS Inc., Chicago, IL). The chi-squared test of significance was utilized to establish statistical correlations between calcium and magnesium levels post-total thyroidectomy. A p-value of less than 0.05 was considered statistically significant.

Results: The study analyzed 100 total thyroidectomy patients. The mean age of patients in our study was 50.7±8.86 years, with 97 females and three males. The most common pathology indicating total thyroidectomy was diffuse colloid goiter (46%), followed by multinodular goiter (38%). Only a single patient had preoperative biochemical hypocalcemia or hypomagnesemia, but none exhibited symptoms. After total thyroidectomy, 15% (n=15) developed hypocalcemia, and 11% (n=11) developed hypomagnesemia. Postoperative mean PTH levels slightly decreased to 28.8±11.75 pg/dl, indicating similar variability to preoperative levels. Patients who underwent intra-capsular dissection had a mean postoperative ionic calcium level of 4.89±0.54 mg/dl, while those who underwent extra-capsular dissection had a slightly lower mean ionic calcium level of 4.72±0.76 mg/dl.

Conclusion: Hypocalcemia is one of the most prevalent complications associated with total thyroidectomy. The role of magnesium in maintaining calcium homeostasis after thyroidectomy should be further explored to improve the management of hypocalcemia. Additionally, the type of capsular dissection performed during the surgery can impact the occurrence of hypocalcemia, and using intra-capsular dissection whenever possible may help reduce the incidence of hypocalcemia.

## Introduction

Thyroidectomy is a surgical procedure performed by general and head and neck surgeons worldwide [1]. The recommended surgical treatment for various thyroid conditions, including both benign (such as colloid goiter and multinodular goiter) and malignant (such as papillary carcinoma and follicular carcinoma) pathologies, has been established as total thyroidectomy. Greater surgical complexity associated with total thyroidectomy correlates with an increased likelihood of postoperative complications, the most prevalent of which is hypocalcemia. The prevalence of postoperative hypocalcemia was as high as 83% with symptoms manifesting in up to 36% of cases [1,2].

Hypocalcemia following thyroidectomy is often caused by hypoparathyroidism, secondary to the unintentional removal of the parathyroid gland or its vascular components. Extensive studies have been conducted throughout the years to comprehend the effects of total thyroidectomy on postoperative calcium levels [3]. However, there is a dearth of research that has specifically examined the relationship between calcium and magnesium levels after surgery.

Levine and Coburn proposed that magnesium has the potential to imitate or counteract calcium by competing with calcium for specific receptors on the parathyroid cell [4]. The administration of intravenous magnesium to patients with hypocalcemia caused by hypomagnesemia resulted in a substantial increase in serum parathyroid hormone (PTH) levels within one minute of the injection, as demonstrated by a study conducted by Rude et al. [5]. Consequently, hypomagnesemia can obstruct the secretion of PTH. Additionally, there is evidence suggesting that hypomagnesemia has a role in the heightened breakdown of PTH.

A comprehensive examination of both calcium and magnesium is crucial for a thorough knowledge of their homeostasis, particularly during the postoperative period, as they have a significant impact on each other's metabolism. The purpose of this study was to highlight the occurrence of hypocalcemia and hypomagnesemia following total thyroidectomy and to assess the correlation between magnesium and calcium levels, thereby providing a better understanding of their homeostasis.

## Materials and methods

This prospective observational research was conducted between 2022 and 2024 at a tertiary care health center. Included in the investigation were 100 consenting study participants undergoing total thyroidectomy at our center. Institutional ethics clearance was obtained from the Dr. D. Y. Patil Vidyapeeth Institutional Ethics Sub-Committee before beginning the study (approval number: I.E.S.C./328/2022). The study was initiated only after obtaining informed consent from all the participants. All referred patients presenting with thyroid pathologies warranting total thyroidectomy were included in the study.

The diagnostic criteria employed for inclusion in the study were based on the presence of clinical/ultrasound/histopathological features indicative of thyroid pathologies including both benign (colloid goiter, multinodular goiter, thyroiditis) and malignant (papillary/follicular carcinoma thyroid). Normal serum calcium levels were estimated at 9-11 mg/dl. Hypocalcemia was defined as serum calcium levels below 8.5 mg/dl. Similarly, normal serum magnesium levels were estimated at 1.7-2.2 mg/dl. Hypomagnesemia was defined as serum magnesium levels below 1.5 mg/dl (all biochemical values were as per our institutional standards).

Patients with pre-existing conditions affecting calcium levels in the body (such as chronic renal failure) were excluded from the study. In addition, patients with medullary carcinoma thyroid were also excluded from the study. All the included participants were evaluated thoroughly using detailed clinical examination and biochemical evaluation (including complete blood count, serum electrolytes, renal and liver function tests, coagulation profile, and thyroid function test), and the findings were logged in dedicated proforma created for this research.

In each group, the preoperative serum calcium levels, preoperative serum magnesium levels, and preoperative serum PTH levels were documented. All patients (n=100) were then subjected to total thyroidectomy (with or without neck dissection in malignant cases) performed under general anesthesia. The decision regarding the type of capsular dissection (intra-capsular or extra-capsular) was based on the pathology (benign/malignant) with malignant lesions requiring exclusive extra-capsular dissection.

The parameters recorded intraoperatively included operative time and volume of intraoperative fluids. Postoperatively, serum calcium and magnesium levels were measured on days 1, 3, 5, 7, 9, and 15. Additionally, intact PTH levels were estimated on days 1 and 15. The occurrence of complications such as hypocalcemia and hypomagnesemia were documented. A comparison was made between pre- and postoperative parameters using the chi-squared test of statistical significance to establish the occurrence of hypocalcemia and the role of magnesium in its maintenance. All patients developing hypocalcemia were treated with measures ranging from oral calcium and calcitriol supplements to intravenous calcium replacement depending on the severity. All patients developing hypomagnesemia were treated with oral followed by intravenous magnesium replacement (in severe cases with symptoms).

The descriptive statistics were computed to delineate the study sample. After completion of data collection, data analysis was achieved using IBM SPSS Statistics for Windows, V. 16.0 (SPSS Inc., Chicago, IL). The associations between preoperative and postoperative values of serum calcium and magnesium were established using the chi-squared test of statistical significance (χ²) at a degree of freedom (dF) of 1. The estimated p-value was considered statistically significant if it was less than 0.05 at a 95% confidence interval. Similarly, the comparison of intra-capsular and extra-capsular dissection in terms of occurrence of postoperative hypocalcemia was also achieved using the chi-squared test of significant (χ²) with the degree of freedom being 1 (dF=1).

## Results

The age group with the highest number of patients undergoing total thyroidectomy in our study was the fifth decade. The mean age group in our study was 50.7±8.86 years (mean±standard deviation). Of the 100 patients, 97 were female and three were male. The observed sex ratio was 33:1 indicating a female predominance (Table 1).

**Table 1:** Age- and gender-wise distribution of study participants

Age groups	Female (%)	Male (%)	Total (%)
31-40	11 (11.34%)	0 (0%)	11 (11%)
41-50	36 (37.11%)	1 (33.33%)	37 (37%)
51-60	35 (36.08%)	1 (33.33%)	36 (36%)
61-70	15 (15.46%)	1 (33.33%)	16 (16%)
Total	97 (97%)	3 (3%)	100% (100%)

The most common pathology encountered in our study indicating total thyroidectomy was diffuse colloid goiter (46%; n=46) followed by multinodular goiter (38%; n=38). Among the malignant etiologies, papillary carcinoma was the most commonly encountered variant (4%; n=4) followed by follicular carcinoma (2%; n=2).

In our study, only a single patient had preoperative biochemical hypocalcemia, i.e., serum calcium <8.5 mg/dl (1%; n=1), whereas the other participants demonstrated normal calcium levels. Similarly, two patients had preoperative biochemical hypomagnesemia, i.e., serum magnesium <2.2 mg/dl (2%; n=2). However, none of the patients exhibited any symptoms of hypocalcemia or hypomagnesemia.

Following total thyroidectomy, the majority of our study participants (i.e., 85%; n=85) did not develop postoperative hypocalcemia, while a small fraction (15%; n=15) developed hypocalcemia. Among the 15 patients who developed biochemical hypocalcemia, only 6% (n=6) of patients developed symptomatic hypocalcemia. The symptoms encountered varied from peri-oral tingling to more serious manifestations such as cardiac arrhythmias (Table 2).

**Table 2:** Incidence of hypocalcemia in the postoperative period Hypocalcemia was defined as serum calcium levels less than 8.5 mg/dl (as per our institutional laboratory standards)

Postoperative hypocalcemia	Frequency (n)	%
Clinical hypocalcemia (symptomatic)	6	40%
Biochemical hypocalcemia (asymptomatic)	9	60%
Total	15	100%

Similarly, 11% (n=11) of patients developed post-thyroidectomy hypomagnesemia, of which only 5% (n=5) presented with symptoms including anorexia, lethargy, and mental confusion. The symptoms encountered varied from peri-oral tingling to more serious manifestations such as cardiac arrhythmias (Table 3). The mean preoperative PTH level was recorded at 29.45±11.86 pg/dl (mean±standard deviation), indicating moderate variability around the mean.

**Table 3:** Incidence of hypomagnesemia in the postoperative period (hypomagnesemia was defined as serum calcium levels less than 1.5 mg/dl) Hypomagnesemia was defined as serum magnesium levels less than 1.5 mg/dl (as per our institutional laboratory standards)

Postoperative hypomagnesemia	Frequency (n)	%
Clinical hypomagnesemia	5	45.45%
Biochemical hypomagnesemia	6	54.54%
Total hypomagnesemia	11	100%

In patients with low preoperative serum calcium (n=1), there were no incidences of postoperative hypomagnesemia. Eleven individuals with preoperative normocalcemia developed post-thyroidectomy hypomagnesemia. On the other hand, the remaining 88 had normal postoperative serum magnesium levels (Table 4). However, the preoperative serum calcium and postoperative serum magnesium levels did not significantly correlate (p=0.761).

**Table 4:** Association of preoperative serum calcium levels with postoperative serum magnesium levels This table presents the analysis of the relationship between preoperative serum calcium levels and postoperative serum magnesium levels in a cohort of 100 patients. The statistical analysis included a chi-squared test of independence to assess the association between the two variables. The chi-squared test statistic was 0.0921, with the degree of freedom as 1. The p-value for this test was 0.761±0.001, which was greater than the level of significance of 0.05. This indicates that there was no statistically significant association between preoperative serum calcium levels and postoperative serum magnesium levels in this sample

Preoperative serum calcium levels	Postoperative serum magnesium levels			Chi-squared test of statistical significance (χ²)	Degree of freedom (df)	P-value
	Low	Normal	Total	0.0921	1	p=0.761
Low	0 (0%)	1 (0.11%)	1 (1%)			
Normal	11 (100%)	88 (98.87%)	99 (99%)			
Total	11 (100%)	89 (100%)	100 (100%)			

Out of the 15% (n=15) patients who had low postoperative serum calcium, 3% (n=3) experienced low postoperative serum magnesium, while the remaining 12% (n=12) had normal postoperative levels. The p-value of 0.218 suggested that there was no significant correlation between postoperative calcium and magnesium levels (Table 5).

**Table 5:** Association of postoperative serum calcium levels with postoperative serum magnesium levels This table presents the analysis of the relationship between postoperative serum calcium levels and postoperative serum magnesium levels in a cohort of 100 patients. The statistical analysis included a chi-squared test of independence to assess the association between the two variables. The chi-squared test statistic was 1.511, with the degree of freedom as 1. The p-value for this test was 0.218±0.002, which was greater than the level of significance of 0.05. This indicates that there was no statistically significant association between postoperative serum calcium levels and postoperative serum magnesium levels in this sample

Postoperative serum calcium levels	Postoperative serum magnesium levels			Chi-squared test of statistical significance (χ²)	Degree of freedom (df)	P-value
	Low	Normal	Total	1.511	1	p=0.218
Low	3 (27.27%)	12 (13.38%)	15 (15%)			
Normal	8 (72.72%)	77 (86.51%)	85 (85%)			
Total	11 (100%)	89 (100%)	100 (100%)			

Out of the patients who had low preoperative serum magnesium (2%; n=2), 2% (n=2) patients experienced low postoperative serum magnesium levels, while none of them had normal postoperative levels. Out of the patients who had normal preoperative serum magnesium levels (98%, n=98), 9% (n=9) experienced low postoperative magnesium levels, while 89% (n=89) maintained normal postoperative levels. According to the provided data, a p-value of less than 0.001 (p<0.05) indicated a significant association between preoperative serum magnesium levels and postoperative serum magnesium levels.

Postoperative mean PTH levels slightly decreased to 28.8±11.75 pg/dl, indicating similar variability to the preoperative levels. The standard error of the mean for postoperative levels was 1.17.

For the patients who had undergone intra-capsular dissection (64%; n=64), the mean postoperative ionic calcium level was 4.89±0.54 mg/dl. On the other hand, patients who had extra-capsular dissection of the thyroid gland (36%; n=36) had a slightly lower mean ionic calcium level at 4.72±0.76 mg/dl after surgery. The p-value was 0.0076, indicating a statistically significant correlation (Table 6).

**Table 6:** Association of postoperative serum calcium levels with the type of ITA ligation The chi-squared test result indicated a statistically significant association between the type of ITA ligation and postoperative serum calcium levels, with a p-value of 0.0076, where the level of significance was p<0.05. The contingency coefficient of 0.258 suggests a moderate association between the type of ligation and the calcium levels. This implies that the method of ITA ligation may influence postoperative serum calcium levels, with extra-capsular ligation being associated with a higher incidence of low calcium levels postoperatively compared to intra-capsular ligation ITA: inferior thyroid artery

Parameters		Type of ITA ligation		Chi-squared test of statistical significance (χ²)	Degree of freedom (df)	P-value
		Intra-capsular ligation of ITA	Extra-capsular ligation of ITA	7.131	1	p=0.0076
Postoperative mean serum calcium levels	Low calcium	5 (7.81%)	10 (25.64%)			
	Normal calcium	59 (92.18%)	26 (66.66%)			
	Total	64 (100%)	36 (100%)			

## Discussion

Thyroidectomy represents a frequently performed surgery by a wide array of surgeons all over the world. Though performed regularly, the procedure demands a significant amount of precision and technical expertise to avoid complications which can significantly alter the postoperative course of the patient [6,7]. Among these dreaded complications, hypocalcemia constitutes the most frequently encountered problem. This complication becomes clinically and biochemically evident between the second and fifth days post-surgery. This hypocalcemia can be transient or permanent and can be the result of more obvious causes such as transient hypoparathyroidism (due to parathyroid injury or vascular compromise during surgery) or due to uncommon factors such as thyroid hormone status, systemic metastasis, etc. [8,9]. Transient hypocalcemia persists for around six months; however, it shows resolution beyond six months, once the parathyroid glands resume function. Permanent hypocalcemia (1.15%) is one that persists beyond six months post-total thyroidectomy. The measurement of intact PTH levels post-surgery provides a valuable insight towards transient hypocalcemia (15-45 pg/ml) [2].

Calcium ion represents an essential element required for performing numerous life-sustaining functions of the body [10]. Numerous factors influence the homeostasis of this element, of which magnesium plays a significant role [11]. Deficiency of the magnesium ion plays a significant role in aggravating post-thyroidectomy hypocalcemia, thus adding to the postoperative morbidity [12,13]. A detailed understanding of both these ions including their interactions in addition to other factors influencing hypocalcemia is essential to deliver appropriate measures to reduce post-thyroidectomy morbidity and consequent mortality.

In line with the studies conducted by Chincholikar and Ambiger, the mean age at presentation in our investigation was recorded as 50.71±8.86 years, with 37 patients being in the age range of 41-50 years [14]. The majority of participants in our study were female (n=97) with a sex ratio of 33:1. The study found that the most commonly observed pathology was colloid goiter, followed by multinodular goiter in 46% and 23% of cases, respectively. These findings shared similarities with the study by Chincholikar and Ambiger [14].

In this study, 15 patients (n=15) demonstrated biochemical hypocalcemia (serum calcium <8.5 g/dl), of which only six patients (n=6) exhibited symptoms. In the same way, 11 patients showed biochemical hypomagnesemia (n=11), of which six individuals (n=6) exhibited symptoms. Poongkodi and Devadas demonstrated that post-thyroidectomy, 31.4% (n=98) of the patients developed hypocalcemia, of which 16.7% (n=52) had persistently low levels of calcium despite treatment [15]. Also, 30.8% (n=96) demonstrated isolated hypomagnesemia, and 15.7% (n=49) had both hypocalcemia and hypomagnesemia. A study conducted by Wilson et al. analyzed the concentrations of calcium and magnesium in 50 persons who had recently undergone total thyroidectomy [16].

In the postoperative phase, 34 patients (78%) experienced hypocalcemia, 36 patients (72%) had hypomagnesemia, and 18 patients (36%) had symptoms. Two of our study participants, who had low preoperative serum magnesium, also experienced postoperative hypomagnesemia. The study revealed that nine patients, who had normal magnesium levels before surgery, experienced a decrease in magnesium levels after the operation, while 89 patients maintained normal magnesium levels postoperatively. Based on the data, it is evident that there is a strong correlation between preoperative serum magnesium levels and the occurrence of postoperative hypomagnesemia. The p-value, which is less than 0.001, further supports this significant association. Chincholikar and Ambiger, unfortunately, did not find any significant association between postoperative hypomagnesemia and preoperative low magnesium levels (p=0.626) [14].

In our study, it was found that only one participant had low calcium levels before undergoing surgery when assessing the association of postoperative hypomagnesemia with preoperative calcium levels. However, the association between postoperative hypomagnesemia and preoperative calcium levels did not reach statistical significance (p=0.761). In a study conducted by Chincholikar and Ambiger, it was found that hypomagnesemia occurred in 17 individuals. However, the results did not show any statistical significance (p=0.152) [14]. Based on these associations, we can assume that magnesium plays a role in the maintenance of calcium homeostasis and justifies the role of magnesium replacement as one of the adjuncts in the treatment of post-thyroidectomy hypocalcemia.

A safe and straightforward surgical procedure, the complete excision of the thyroid gland necessitates meticulous dissection to protect the parathyroid gland and reduce the likelihood of hypocalcemia following the procedure [17,18]. According to R and Panduranga Rao, the main goal of intra-capsular dissection should be to split only the third-level branches of the inferior thyroid artery and move on to the back part of the neck [19]. As a result, the parathyroid gland's vascular supply is typically well-maintained. This is advantageous because it can reduce the likelihood of postoperative hypocalcemia and hypomagnesemia, thereby reducing the likelihood of surgical complications. Rageh et al. conducted a prospective study on 50 patients who underwent intra-capsular complete thyroidectomy and found that none of the patients experienced any complications, including recurrent laryngeal nerve (RLN) palsy or compromise of the parathyroid glands [20].

The study was subject to certain limitations, namely, a small sample size, a lack of measures taken to avoid bias, and a limited duration of follow-up. Nevertheless, we intend to further our research by incorporating a large study cohort, thus yielding more comprehensive data that can contribute to the vast ocean of data on post-thyroidectomy hypocalcemia.

## Conclusions

Hypocalcemia is a detested complication of total thyroidectomy that, if not promptly addressed, can result in a substantial financial burden and a detrimental impact on the patient's quality of life. We still lack a detailed understanding of magnesium's role in the occurrence and management of post-thyroidectomy hypocalcemia. The present study has attempted to shed light on the role of magnesium in maintaining post-thyroidectomy calcium homeostasis. Total thyroidectomy was associated with a relatively low occurrence of hypocalcemia and hypomagnesemia in our study sample. Furthermore, the associations between preoperative serum calcium-postoperative serum magnesium and postoperative serum calcium-postoperative serum magnesium, though statistically insignificant, demonstrated a direct or indirect role of magnesium in stabilizing serum calcium levels post-total thyroidectomy. The majority of patients who experienced hypocalcemia or hypomagnesemia exhibited solely positive laboratory results, whereas only a small number displayed symptoms. Furthermore, intra-capsular dissection demonstrated a decreased occurrence of hypocalcemia in comparison to extra-capsular dissection. Therefore, whenever possible, the adoption of intracapsular dissection can aid in reducing the incidence of hypocalcemia resulting from parathyroid devascularization.
